# Regional Outcome Evaluation Program (P.Re.Val.E.): Reduction of inequality in access to effective health care in the Lazio region of Italy (2012–2015)

**DOI:** 10.1371/journal.pone.0194972

**Published:** 2018-03-27

**Authors:** Martina Ventura, Danilo Fusco, Katia Bontempi, Paola Colais, Marina Davoli

**Affiliations:** Department of Epidemiology of Lazio Regional Health Service, Rome, Italy; Universita degli Studi di Ferrara, ITALY

## Abstract

**Background:**

Inequalities in health among groups of various socio-economic status (as measured by education, occupation, and income) constitute one of the main challenges for public health. Since 2006, the Lazio Regional Outcome Evaluation Program (P.Re.Val.E.), presents a set of indicators of hospital performance based on quality standards driven by strong clinical recommendations, and measures the variation in the access to effective health care for different population groups and providers in the Lazio Region. One of the aims of the program was to compare population subgroups in order to promote equity in service provision. Since June 2013, a new management strategy has been put in place that assigned specific goals based on performance assessment to the chief executive officers of the hospitals.

**Aim:**

To evaluate whether, in recent years, there has been a reduction in the differential access to effective health care, among individuals with different educational levels.

**Methods:**

We enrolled all patients discharged from both public and private hospitals of the Lazio region between 2012 and 2015, living in Lazio region. We analysed the proportion of patients with ST-elevation myocardial infarction (STEMI) treated with percutaneous coronary intervention within 90 minutes (primary PCI), the proportion of patients with hip fracture (HF) who underwent surgery within 2 days, and the proportion of women with primary C-section. We applied multivariate logistic regression models to assess the effect of educational level on health outcomes, adjusting for demographic characteristics and comorbidities that could affect the outcomes. For each year of the study period, we compared adjusted proportions of outcomes for the highest and the lowest level of education by using percentage differences.

**Results:**

In the Lazio region, 44.6% of STEMI patients (N = 3,299) were treated with primary PCI, 54.4% of patients with hip fractures (N = 6,602) underwent surgery within 2 days, and 27.7% of women without a previous C-section (N = 34,718) delivered via C-section, in 2015. The corresponding proportions in 2012 were 27.8%, 31.3% and 31.5%, respectively. By comparing the adjusted proportions in patients with the highest education level (a university degree or higher) to those with the lowest level education level (None/Primary school), a decrease in the percentage difference was observed during the study period. In STEMI and delivery cohorts, the improvement of outcomes involved the least and the most educated patients, respectively, and the difference between the two educational levels was close to zero in 2015, whereas for hip patients, the improvement was more evident among the less educated patients.

**Conclusions:**

In the Lazio region, we observed a reduction in the differential access to effective heath care by educational level, in different clinical areas. Different factors might explain these results. On top of the public disclosure of outcome data, the management strategy applied in mid-2013 might have driven the overall improvement of the health system for the considered conditions, helping to achieve a fairer access to health.

## Background

Access to health care should be equitable and ensured to all citizens regardless of their personal characteristics, such as gender, age, race, ethnicity and socioeconomic status—SES [1, 2]. Horizontal inequality is seen as a major limitation to improving population health [3].

The relationship between socioeconomic position and health is complex and influenced by various factors, including the characteristics of health care systems. Health inequalities can be attributed to higher vulnerability of disadvantaged social segments of population [4], to a greater susceptibility of those, to risky health behaviours and to unequal access to effective health care.

Health care disparities have been highlighted in many countries with different health care systems [5–7]. In Italy, where the National Health Service (NHS) provides universal health coverage, economic or social barriers are not expected; yet, inequalities in obtaining optimal care have been reported [3, 8–11].

The association between SES and health outcomes has been extensively investigated, both in the general population and in specific patient groups [12–14]. Educational level has been widely used as an individual proxy of SES [13, 15, 16]. Indeed, educational attainment should be considered as a determinant of future income and occupation, capturing aspects related to the social opportunities of individuals [13, 15, 17]. It also provides knowledge and life skills that allow better-educated persons to gain more ready access to information and resources to promote health [18].

In Italy, since 2006, the Lazio Regional Outcome Evaluation Program (P.Re.Val.E.) has measured adherence to quality standards among hospital and population groups using a set of indicators driven by strong clinical recommendations [19]. One of the aims of the program was to compare population subgroups (e.g. socioeconomic subgroups) in order to evaluate and promote equity in service provision. In the “Equity section” of P.Re.Val.E., a set of well-established indicators are presented in order to analyse the association between health outcomes and educational level. Such an evaluation program has been publicly available since 2007 [20], and in June 2013, a management strategy based on this program was put in place. In fact, since 2013, some indicators that were defined and calculated in P.Re.Val.E. have become part of the set of indicators used to measure and monitor the performance of the chief executive officers (CEOs) of hospitals in the Lazio Region. Threshold values of those indicators were used to measure the achievement of goals and were part of an overall performance evaluation system used to confirm the mandate of the CEO.

There is already evidence of the effectiveness of public disclosure of performance data, and regulatory or pay for performance interventions in improving access to effective health care and health outcomes [21–25].

However, only a few studies have demonstrated whether an overall improvement in the quality of care has had a differential impact on different subgroups of the population [26, 27].

Therefore, the aim of this study was to evaluate whether, in recent years, there has been a reduction in the differential access to effective health care, among individuals with different educational levels, by using some indicators defined in P.Re.Val.E. that belong to different clinical areas, and were chosen as goals for the CEOs of Lazio Region hospitals.

## Methods

### Data sources

Data were collected using the regional Hospital Information Systems (HIS) database that includes discharge abstracts with socio-demographic data (gender, age, place of residence, educational level) and clinical information (diagnoses, procedures), for all hospital admissions of Lazio region. Additional information on comorbidities and time to surgery was derived by the Emergency Information System (EIS) and from the Admission and Discharge Information System (RAD Esito). RAD Esito collects supplementary clinical data on acute myocardial infarction (AMI) and hip fractures since 2008, such as systolic blood pressure at admission for patients with a diagnosis of AMI, the pre-operative creatinine level and the value of the International Normalised Ratio (INR) for patients with hip fracture. Data from different information systems were linked using a deterministic record-linkage procedure based on anonymous identification codes.

### Study population

We enrolled all patients discharged from any hospital of the Lazio region between January 1^st^, 2012 and December 31^th^, 2015 and living in the Lazio region.

We focused on three patient cohorts representing common conditions, as shown in previous studies, to achieve stable estimates [28].

We included patients with ST-elevation myocardial infarction (STEMI), patients admitted for hip fracture, and women who had given birth during the study period. In the STEMI cohort, we considered all patients older than 18 years, discharged with a main diagnosis of AMI (ICD-9-CM 410) or with a main diagnosis of a condition compatible with myocardial infraction, and with AMI in the secondary diagnosis. We excluded all AMI episodes with at least a diagnosis of No STEMI (ICD-9-CM 410.7 or 410.9).

The hip fracture cohort consisted of all ordinary admissions of patients over 65 years of age, with a diagnosis of hip fracture (ICD-9-CM 820.0–820.9).

All deliveries were selected using diagnosis-related groups (DRGs), diagnosis, and procedure ICD-9-CM codes. Women aged 10–55 years old without a previous caesarean section were included.

More detailed information on the inclusion and exclusion criteria used for the selection of the cohorts are available on the P.Re.Val.E. web site [20].

### Outcomes

We considered the outcomes used for the evaluation of achievement of the CEOs’ mandate and budget goals. Thus, the following indicators were included:

- the proportion of patients with STEMI treated with percutaneous coronary intervention (PCI) within 90 minutes (primary PCI);
- the proportion of interventions within 2 days in patients over 65 years of age with hip fracture;
- the proportion of women who underwent primary C-section.

### Exposure

To evaluate whether there was a reduction of inequalities in access to effective health care, we considered as exposure the educational level of the patients.

According to a previous empirical analysis [29] in which the authors tested the agreement between the educational level from the HIS and that recorded in the 2001 Census, the information was more reliable when retrieved by an elective hospital discharge than by an urgent one. Since we had all the discharge abstract of our patients, we selected the most reliable information, according to the findings of the previous work. Therefore, if the index admission was urgent, we looked at other elective hospitalizations (up to one year before the index admission) of the same patient. If we found a different educational level, we used the information recorded on the elective hospitalization.

### Statistical analysis

We used multivariate regression analysis to assess the effect of educational level on outcomes, accounting for patient demographic characteristics and comorbidities that could affect the outcome under study.

Among all factors potentially associated with the outcome (S1 Appendix), age and gender were considered a priori risk factors, apart from the delivery cohort. The other factors were selected using a stepwise bootstrap procedure to assign an importance rank of predictors in the logistic regression analysis. In this approach, we ran the logistic regression with all predictors 100 times on random samples drawn with replacement from the original data set. Only risk factors that were identified at least 30 times as being significant (p<0.05) were included in the risk adjustment model.

To estimate the annual adjusted proportion of outcome for each indicator, we applied a multivariate logistic regression analysis with no intercept, including centred covariates and an interaction term. This model estimates the log odds of the outcome by educational level.

Adjusted proportions were obtained for each level of exposure by back-transforming the parameter estimates with the following formula:

Adjproportion=[exp(estimate)/(1+exp(estimate))]*k

where k is a correction coefficient, introduced to account for the nonlinear nature of the logistic model [19].

We carried out the time trend analysis from 2012 to 2015 by including in the adjustment model an interaction term between exposure (educational level) and year of analysis. Thus, the complete comparability among the adjusted estimates obtained for different years was ensured.

For each year of the study period, we compared adjusted proportions of outcomes for the highest and the lowest level by using percentage differences.

All analyses were undertaken using SAS Version 9.2.

### Secondary analysis

In order to evaluate whether the presence of misclassification of exposure could affect the results of our study, we carried out a secondary analysis using as exposure an area-based SES index [13]. This index was calculated for the census blocks of Lazio region using the 2011 Census data and considering various dimensions of deprivation: education, occupation, housing tenure, family composition and immigration.

## Results

Every year, approximately 1 million hospital discharge records are collected in the regional health information systems of the Lazio region; of these patients, approximately 90% also lived in the region. Between 2012 and 2015, we found 3,608,033 hospitalizations in regional facilities (both public and private) of people living in Lazio.

We considered three cohorts of patients with different characteristics. After applying all the selection criteria, in the study period, we enrolled 14,327 patients with ST-elevation myocardial infarction (mean age 67, 29.8% females), 27,964 patients with a hip fracture (mean age 83, 76.8% females), and 157,928 deliveries (mean age 32).

**Fig 1:**
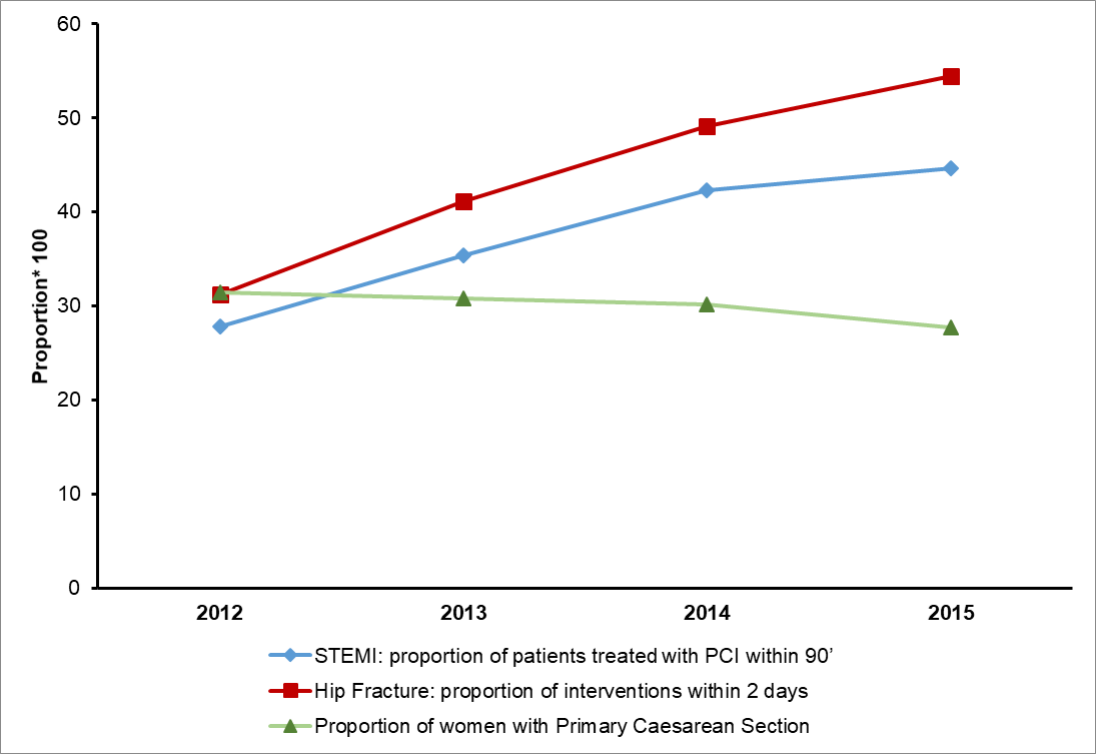
Proportions of outcomes, Lazio region 2012–2015.

In 2012, the proportion of STEMI patients (N = 3,761) treated with PCI within 90 minutes was 27.8%, the proportion of hip fractures (N = 7,166) operated on within 2 days was 31.3%, and the proportion of women (N = 42,997) without a previous C-section who delivered with a C-section was 31.5%. During the study period, all the outcome measures improved (Fig 1). A considerable increase was observed for primary PCI among STEMI and early interventions for hip fractures that were 44.6% and 54.4%, respectively, in 2015, and a decrease of up to 27.7% was found in the proportion of primary C-section cases.

Table 1 shows the association between the three different outcomes and educational level at the beginning and at the end of study period. In 2012, a significantly higher probability of being treated with primary PCI (RR = 1.80 p<0.001), and of being operated on within 2 days for hip fractures (RR = 1.87 p<0.001), was found when comparing patients with a graduate degree (“University degree”) to those with the lowest level of education (“None/ Primary school”). For C-section deliveries, a higher education level was associated with a higher risk of having a C-section (RR = 1.19 p<0.001).

**Table 1:** Association between outcomes and educational level at the beginning and end of the study period (2012 and 2015).

		2012					2015				
	Educational level	N	Crude prop. (%)	Adj prop. * (%)	RR	p-value	N	Crude prop. (%)	Adj prop. * (%)	RR	p-value
**STEMI: proportion of patients treated with PCI within 90'**	***None/Primary school***	1169	19.8	24.5	1		1011	37.6	43.4	1	
	***Middle school***	1399	28.9	27.1	1.11	0.167	1183	46.2	45.2	1.04	0.437
	***High school***	992	32.6	28.9	1.18	0.032	929	50.2	46.9	1.08	0.156
	***University degree***	191	45.0	44.2	1.80	<,001	176	45.5	43.4	1.00	0.994
**Hip fracture: proportion of intervention within 2 days**	***None/Primary school***	4535	25.7	25.4	1		3844	49.8	49.7	1	
	***Middle school***	1619	39.1	39.2	1.55	<,001	1581	58.4	59.0	1.19	<,001
	***High school***	750	43.5	44.1	1.74	<,001	954	64.1	64.6	1.30	<,001
	***University degree***	250	45.6	47.6	1.87	<,001	215	65.6	67.1	1.35	<,001
**Proportion of women with primary c-section**	***None/Primary school***	2616	27.8	27.7	1		2903	26.6	27.2	1	
	***Middle school***	10094	29.4	31.1	1.12	0.004	7742	26.4	28.6	1.05	0.199
	***High school***	20300	31.6	32.1	1.16	<,001	15635	27.4	27.7	1.02	0.598
	***University degree***	9700	34.3	32.9	1.19	<,001	8402	29.9	28.1	1.03	0.381

*Adjusted for

- STEMI: age, gender, previous myocardial infarction, heart failure, cerebrovascular disease, chronic renal disease, systolic blood pressure at admission.

- Hip fracture: age, gender, diabetes, anemias, other forms of ischemic heart disease, conduction disturbances and arrhythmias, cerebrovascular disease, chronic renal disease.

- Deliveries: maternal age, maternal citizenship, cancer, liver disorders in pregnancy, cardiovascular diseases in pregnancy, Antepartum hemorrhage/abruptio placentae/ placenta previa, Pre-eclampsia/eclampsia, preterm labor, multiple pregnancy, fetopelvic disproportion/excessive development of the infant, fetal abnormality, fetal distress, intrauterine growth retardation, Polyhydramnios/ oligohydramnios/ infection of the amniotic cavity, premature rupture of membranes, cord prolapse, hiv, assisted fertilization.

In 2015, we observed no differences anymore in terms of educational level in access to primary PCI among STEMI patients and access to primary C-section among women. However, a significantly higher probability of undergoing an operation within two days was still noted for more educated hip fracture patients (“University degree” versus “None/Primary school”, RR = 1.35 p<0.001), although the relative effect was smaller in 2015 than in 2012.

**Fig 2:**
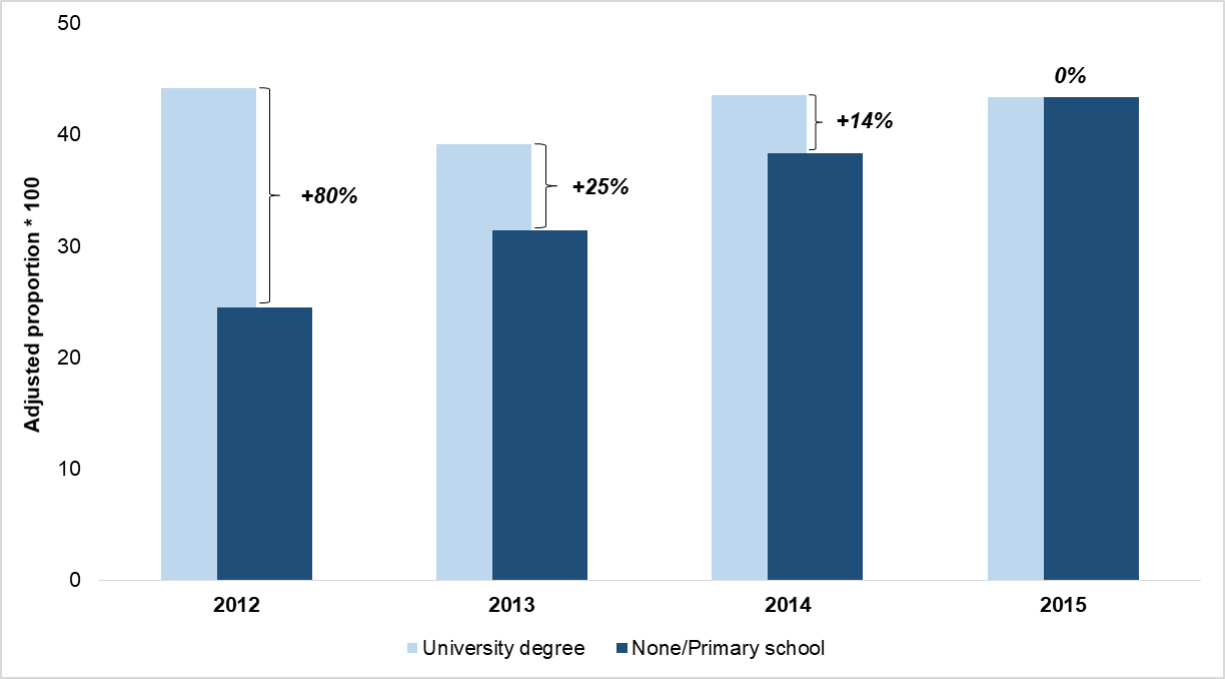
STEMI: Proportion of patients treated with PCI within 90'. Adjusted proportions for the lowest and highest educational levels and percentage differences, 2012–2015.

**Fig 3:**
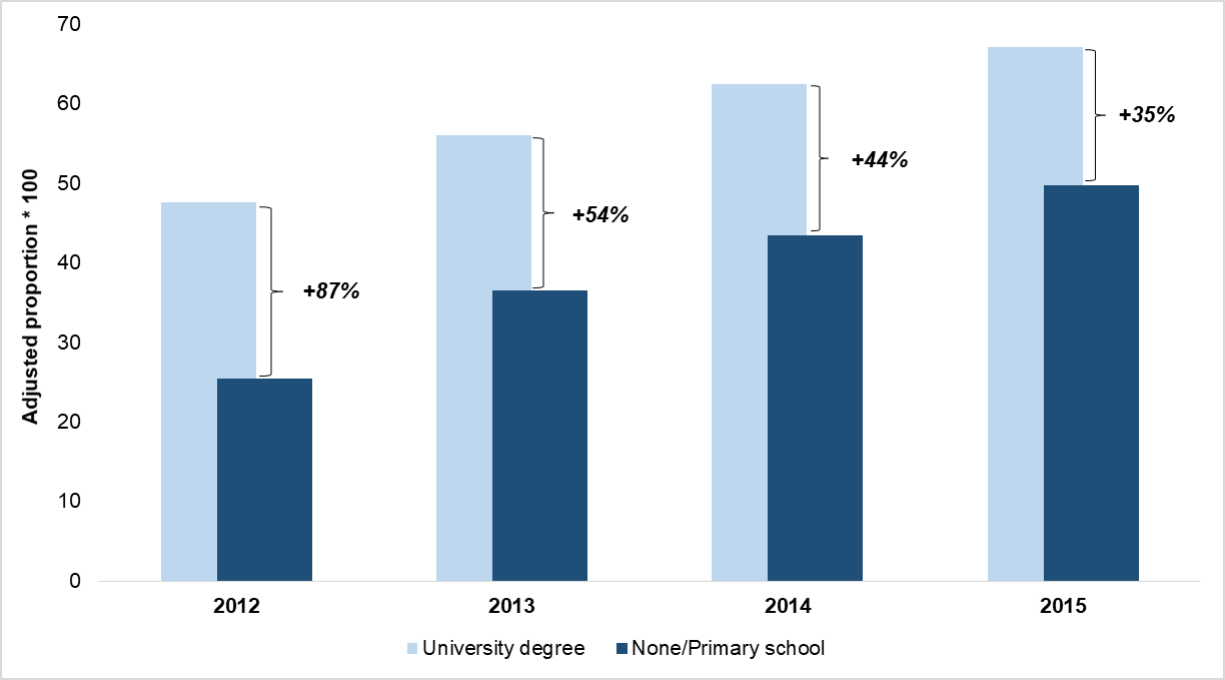
Hip fracture: Proportion of intervention within 2 days. Adjusted proportions for the lowest and highest educational levels and percentage differences, 2012–2015.

**Fig 4:**
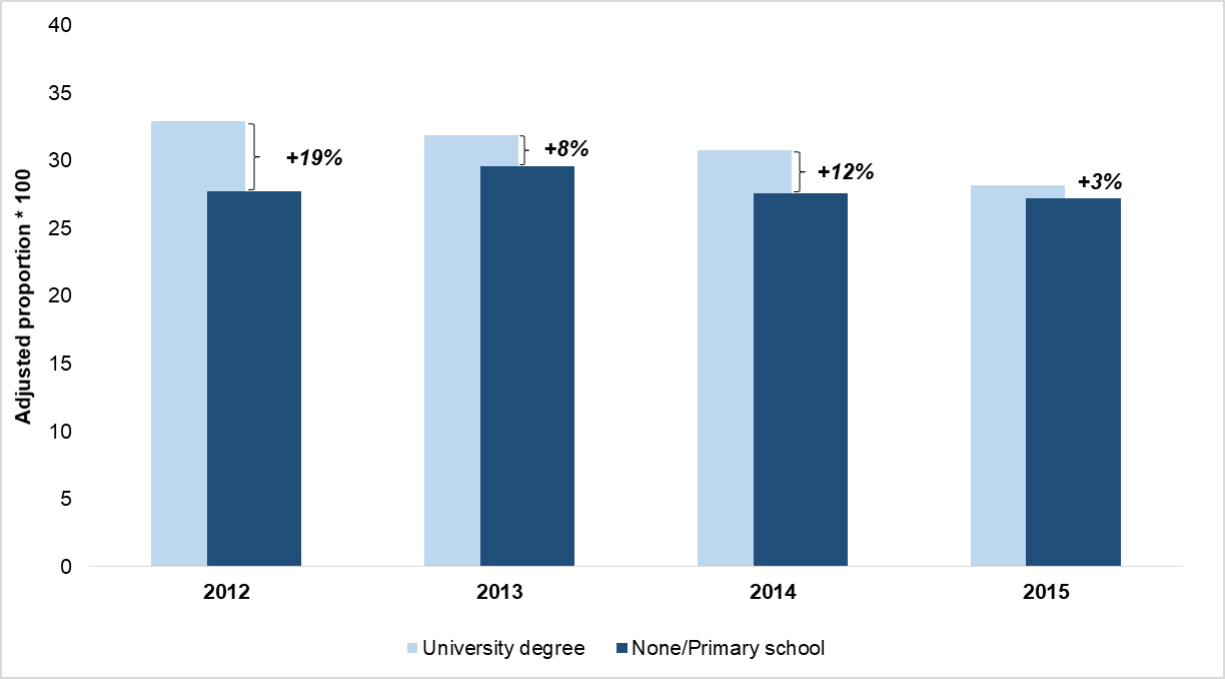
Proportion of women with primary caesarean section. Adjusted proportions for the lowest and highest educational levels and percentage differences, 2012–2015.

Figs 2–4 display the time trend of the comparison between adjusted proportions estimated for the highest and lowest educational level for STEMI, hip fractures and deliveries, respectively. For STEMI patients, the adjusted proportion of primary PCI was substantially unchanged in the patients with a graduate degree, whereas it rose from 24.5% to 43.4% for the least educated patients, bringing the relative difference from 80% in 2012 to zero in 2015. For hip fracture patients, the improvement was more evident among those with the lowest educational level, but it also involved those with the highest educational level, thus reducing the percentage difference from 87% to 35%. Conversely, the major reduction in access to primary C-sections was observed among the most educated women. In 2015, a difference of only 3% remained between the proportion of C-section in the highest and the lowest level of education, and this was mainly due to the decrease of outcome for women with a “University degree” (from 32.9% in 2012 to 28.1% in 2015).

The secondary analysis carried out with the SES index as exposure largely confirmed the main results. For STEMI and hip fracture patients, we observed a significant difference of outcomes between those with low and high SES in 2012, but this difference disappeared in 2015 for the former and reduced in 2015 for the latter. For the deliveries cohort, no significant differences for SES were observed in the probability of having C-section in both 2012 and 2015 (S1 Table).

## Discussion

In this study, we observed an overall improvement of clinical outcomes and a reduction in the differential access to effective health care by educational level in the Lazio region from 2012 to 2015 in three relevant clinical areas.

Since 2012, we observed a significant increase in the proportion of STEMI patients treated with primary PCI, as well as the proportion of hip fractures that were operated on within 2 days. In contrast, we also observed a slight decrease of the proportion of women who underwent primary C-section. These results for patients hospitalized in the Lazio region are consistent with those found at the national [30] and international [31–34] levels, suggesting efforts towards a greater guidelines adherence [35–39].

To our knowledge, this is the first study to investigate whether an average improvement was achieved to benefit the least educated part of the population. Overall, we noted a decrease in the gap between the highest and the lowest educational level. This decrease was attributed to three different scenarios as follows: improvement for the total benefit of the less educated patients (for primary PCI), improvement for both educational levels, although more evident for the lowest level, (for hip fracture), and eventually, improvement only for the more educated women for C-Section.

In patients hospitalized for STEMI, the proportion of graduated patients treated with primary PCI was already above 40% in 2012, and remained substantially unchanged in the study period. Instead, we observed a considerable improvement among the least educated patients, which helped to fill the equity gap in terms of access to effective treatment attributable to educational level. Cacciani et al. [27] had already reported a similar result after observing a decreasing educational differential in the access to PCI between 2001 and 2011, using data from a cohort of STEMI residents in Rome (Italy). This finding seems to suggest that the efforts of the health system in improving health care quality could also be effective in improving equity.

We observed a relatively different scenario for patients with hip fracture. In this case, we found improved access to timely surgery for both the least and the most educated patients, although the increase was much higher among the least educated patients, thus reducing the relative difference. However, inequalities in access to effective treatment were still present at the end of the study period. One possible explanation for this observed gap is the high heterogeneity among hospitals, with some that reached very high standards (more than 80% operated within two days) and others that were far below the minimum standard (less than 10%). The latter hospitals were mainly located outside the metropolitan area of Rome. The effect of such heterogeneity reflects in a failure in reaching the least educated part of the population [20]. In fact, in the Lazio region, the educational level of residents is not uniform among geographical areas, and a higher concentration of the population with a low education level is present outside the city of Rome. Since the delay in hip fracture surgery is significantly associated with an increased risk of mortality and morbidity, initiatives to reduce the waiting times for surgery have been encouraged in Italy and other countries [23]. In the Lazio region, efforts towards a better adherence to guidelines have been put in place, namely, the definition of a specific pathway of care, the definition of a pay for performance scheme and, eventually, the identification of specific goals for the CEOs of the regional hospitals.

The findings on caesarean sections are particularly interesting. Rates of C-sections have increased in nearly all OECD countries, although in a few countries this trend has reversed at least slightly in the past few years [34]. Italy is one of the European countries with the highest annual number of C-sections, but recently, the proportion of women to undergo primary C-section has been slowly decreasing [30], and we observed the same trend in the Lazio region. Inequalities in access to C-section go the other way round. Thus, women in higher socioeconomic positions, regardless of their obstetric risk, have more access to caesarean sections relative to women with lower socioeconomic status. [40, 41]. In our study, the adjusted proportion of C-sections was higher in women with graduate degrees than in those with lower levels of education in 2012, but this difference disappeared by the end of the study period. The improvement concerned the most educated women, for which the proportion decreased to the level of women with low education.

The reduction in C-sections, as well as the achievement of a more equitable situation, could partly be explained by the effort towards the adoption of standardized protocols of obstetric care. Women’s requests for C-section are often reported as being a major determinant of C-section. These findings seem to show that women, and particularly well-educated women, can change their behaviour if properly informed. However, the women’s requests, their ability to address the physician’s decision, and the providers’ economic and organizational interests must certainly play a decisive role.

Therefore, faced with a general improvement of the quality of care, which covered all the procedures considered, condition-specific mechanism should be taken into account when analysing the reduction of inequalities. As we observed, the decrease in the gap between the highest and the lowest educational level was more consistent for STEMI patients, whereas it was more limited for those with hip fracture and for women who delivered. This could suggest a sort of urgency gradient: the greater is the urgency, the more the health care improvement concerns the whole population, and consequently, the more the inequality decreases. Moreover, if for STEMI and hip fracture the health care quality plays the key role, in the C-section the women’s will as well as the providers’ interests have certainly to be considered as important determinants that cannot be measured. Eventually, for the emergencies, health care organisation seems to be more important that patients’ choice.

Different factors may explain the observed improvement in both outcome and equity of health care.

The public release of hospital performance data has been recommended as one key strategy for stimulating improvements in quality of care by putting the focus on the transparency and accountability of health care providers [42]. There is considerable debate regarding public reporting of performance data and its potential effects [43–45]. However, a recent systematic review conducted by Campanella et al. on the impact of public reporting of clinical outcomes concluded that it could stimulate providers to improve healthcare quality, although few data are available on the effect of public reporting on specific population subgroups [21]. In 2012, Renzi et al. showed the positive but limited impact of public reporting of 2006–2009 performance data from the P.Re.Val.E. program in the Lazio region, suggesting that integrated interventions could achieve a greater effect [25].

In addition to the dissemination of the P.Re.Val.E. results, since 2013, some indicators have been used as instruments to monitor the performance of CEOs of Lazio Local Health Authorities and hospitals, requiring them to reach threshold values of outcomes as budget or mandate goals. In addition, an active programme for auditing the data quality has been promoted both at the regional and national level. All these activities could have contributed in stimulating a quality improvement of the health care offer. However, we are not able to identify how much these aspects singularly contribute.

Tackling healthcare inequalities is a priority of the health policy agenda for developed and developing countries [46]. Despite evidence of inequalities in health care in Italy, no systematic national programmes have been established to specifically address the differential in care access and outcomes attributable to different socioeconomic positions [26]. The most recent national regulatory actions do use quality standards, such as those measured by the indicators analysed in this study, as a pay for performance instrument. Moreover, there is a great emphasis, especially at the national level, towards public reporting of performance data. Although our analysis suggests that an overall improvement in the quality of care can also produce a reduction of the equity gap, the continuous monitoring of its impact on equity is necessary.

The strengths of this study include the availability of routinely collected data, retrieved from several linkable information systems, the use of a validated algorithm for cohort selection and variable definition and the robust outcomes. Furthermore, the use of educational level as a proxy of SES allowed us to assess the exposure at individual level, thus avoiding the ecological fallacy of area-based measures. In the Lazio region, the presence of missing data for exposure assessment was not significant: in 2012–2015, only 0.01% of missing values on education was found on the regional HIS. Moreover, the availability of all discharge abstracts allows for the selection of the most reliable information [29]. Nevertheless, some disadvantages have to be considered when using education from HIS as a proxy of SES. First of all, the presence of a certain degree of misclassification of the educational level recorded on the discharge abstracts cannot be ruled out. In addition, although the educational level proved to be a good proxy for SES, we could not consider SES variables other than education, nor the interactions among them, which could provide a more comprehensive picture of individual levels of SES. However, we obtained similar results using the SES index in the secondary analysis.

Some of the study's strengths may simultaneously represent some of its limitations. First, this study was based on HIS; thus, despite some important advantages in collecting administrative data [19, 47], there remains questions about its accuracy, completeness and possible gaps in clinical information [48]. The use of routinely collected administrative data in comparative outcome evaluations has been criticized for the following reasons. There is an absence of clinical information needed to adequately adjust for patients' conditions. Furthermore, some chronic comorbidities, such as hypertension and diabetes, are known to be currently under-reported at admission, mainly in more severely affected patients. The first problem could be partially overcome, since some clinical information (e.g., systolic blood pressure, ejection fraction, creatinine) have been recorded in RAD Esito [49, 50]. The problem of under-recording is also solvable by using prior patient hospitalization records to identify comorbidities independent of patient severity at the current admission, as well as emergency department visits, to collect additional information about patient risk factors [19].

Lastly, even though the possibility of gaming the data in response to the performance evaluation cannot be ruled out, previous studies did not find evidence of gaming [28]. Some studies have reported that changes in data accuracy may partially explain quality improvement [51]. However, relevant changes in recording co-morbidities in our study population over the years were not found. In agreement with previous reports, the prevalence of certain co-morbidities and risk factors was relatively low in our study population, indicating underreporting of co-morbidities and detailed clinical information in the administrative database [19]. However, underreporting was non-differential in the years included in our analysis and by educational level.

In conclusion, our analysis in the Lazio region suggests that different strategies accompanying the public disclosure of performance data may be successful in both improving quality of care and reducing inequalities in the access to effective treatment.

## Supporting information

S1 Appendix List of risk factors used for risk adjustment. (DOCX)

S1 Table Association between outcomes and socio economic status (SES) at the beginning and end of the study period (2012 and 2015). (DOCX)
