# Supplementary material for: Regional Outcome Evaluation Program (P.Re.Val.E.): Reduction of inequality in access to effective health care in the Lazio region of Italy (2012–2015)
Source: PLoS One. 2018 Mar 27;13(3):e0194972. doi: 10.1371/journal.pone.0194972 (PMC5870986; doi:10.1371/journal.pone.0194972)
Supplement: S1 Appendix — (DOCX) [file pone.0194972.s001.docx]

**Appendix. List of risk factors used for risk adjustment**

**1. STEMI: proportion of patients treated with PCI within 90'.**

| **Risk factor** | **ICD-9-CM code** | |
| --- | --- | --- |
|  | **Episode of STEMI** | **Previous 2 years** |
| Age |  |  |
| Gender  Citizenship |  |  |
| Systolic Blood Pressure at admission |  |  |
| Cancer | 140.0–208.9, V10 | 140.0–208.9, V10 |
| Diabetes |  | 250.0-250.9 |
| Lipid metabolism disturbances |  | 272 |
| Obesity | 278.0 | 278.0 |
| Anemias | 280-284, 285 (excl. 285.1) | 280-284, 285 (excl. 285.1) |
| Coagulation defects | 286 | 286 |
| Other hematological diseases | 287-289 | 287-289 |
| Hypertension |  | 401-405 |
| Previous myocardial infarction | 412 | 410, 412 |
| Other forms of ischemic heart disease |  | 411, 413, 414 |
| Heart failure |  | 428 |
| Ill-defined descriptions and complications of heart disease |  | 429 |
| Rheumatic heart disease | 393-398 | 391, 393-398 |
| Cardiomyopathy | 425 | 425 |
| Acute endocarditis and myocarditis |  | 421, 422 |
| Other heart conditions | 745, V15.1, V42.2, V43.2, V43.3, V45.0 | 745, V15.1, V42.2, V43.2, V43.3, V45.0 |
| Conduction disturbances and arrhythmias |  | 426, 427 |
| Cerebrovascular disease | 433, 437, 438 | 430-432, 433, 434, 436, 437, 438 |
| Vascular disease | 440-448 (excl. 441.1, 441.3, 441.5, 441.6, 444) , 557.1 | 440-448, 557 |
| Chronic obstructive pulmonary disease (COPD) |  | 491-492, 494, 496 |
| Chronic renal disease | 582-583, 585-588 | 582-583, 585-588 |
| Chronic diseases (liver, pancreas, intestine) | 571-572, 577.1-577.9, 555, 556 | 571-572, 577.1-577.9, 555, 556 |
| Previous coronary artery bypass graft | V45.81 | 36.1, V45.81 |
| Previous coronary angioplasty | V45.82 | 00.66, 36.0, V45.82 |
| Cerebral revascularization procedures |  | 00.61, 00.62, 38.01, 38.02, 38.11, 38.12, 38.31, 38.32 |
| Other cardiac operations |  | 35, 37.0, 37.1, 37.3, 37.4, 37.5, 37.6, 37.9 |
| Other vascular operations |  | 38-39.5, (excl. 38.01, 38.02, 38.5, 38.11, 38.12, 38.31, 38.32) |

**2. Hip fracture: proportion of intervention within 2 days.**

| **Risk factor** | **ICD-9-CM code** | |
| --- | --- | --- |
|  | **Index hospitalization** | **Previous 2 years** |
| Age |  |  |
| Gender  Citizenship |  |  |
| Diabetes |  | 250.0-250.9 |
| Nutritional deficiencies | 260-263, 783.2, 799.4 | 260-263, 783.2, 799.4 |
| Obesity | 278.0 | 278.0 |
| Anemias | 280-284, 285 (excl. 285.1) | 280-284, 285 (excl. 285.1) |
| Coagulation defects | 286 | 286 |
| Other hematological diseases | 287-289 | 287-289 |
| Dementias including Alzheimer’s disease | 290.0-290.4, 294.1, 331.0 | 290.0-290.4, 294.1, 331.0 |
| Parkinson’s disease | 332 | 332 |
| Hemiplegia and other paralytic syndromes | 342, 344 | 342, 344 |
| Hypertension |  | 401-405 |
| Previous myocardial infarction | 412 | 410, 412 |
| Other forms of ischemic heart diseases |  | 411, 413, 414 |
| Heart failure |  | 428 |
| Ill-defined descriptions and complications of heart disease |  | 429 |
| Rheumatic heart disease | 393-398 | 391, 393-398 |
| Cardiomyopathy | 425 | 425 |
| Acute endocarditis and myocarditis |  | 421, 422 |
| Other heart conditions | 745, V15.1, V42.2, V43.2, V43.3, V45.0 | 745, V15.1, V42.2, V43.2, V43.3, V45.0 |
| Conduction disorders and arrhythmias |  | 426, 427 |
| Cerebrovascular disease | 433, 437, 438 | 430-432, 433, 434, 436- 438 |
| Vascular disease | 440-448 (excl. 441.1, 441.3, 441.5, 441.6, 444), 557.1 | 440-448, 557 |
| Chronic obstructive pulmonary disease (COPD) |  | 491-492, 494, 496 |
| Chronic renal disease | 582-583, 585-588 | 582-583, 585-588 |
| Other chronic disease (liver, pancreas, intestine) | 571-572, 577.1-577.9, 555, 556 | 571-572, 577.1-577.9, 555, 556 |
| Rheumatoid arthritis and other inflammatory polyarthropathies | 714 | 714 |

**3. Proportion of women with primary C-section.**

| **Risk factor** | **ICD-9-CM code** | |
| --- | --- | --- |
|  | **During hospitalization for childbirth** | **Previous 2 years** |
| Maternal age |  |  |
| Maternal citizenship |  |  |
| Cancer | 140.0–208.9, V10 | 140.0–208.9, V10 |
| Anemias | 280-284, 285 (excl. 285.1), 648.2 (excl. 648.22, 648.24) | 280-284, 285 (excl. 285.1) |
| Coagulation defects | 286 | 286 |
| Heart diseases | 390-398, 410-429 | 390-398, 410-429 |
| Cardiovascular diseases in pregnancy | 648.5, 648.6 |  |
| Congenital anomalies of heart and circulatory system | 745-747 | 745-747 |
| Cerebrovascular disease | 433, 437, 438 | 430-432, 433,434,436,437,438 |
| Nephritis, nephritic syndrome and nephrosis | 580-589 | 580-589 |
| Unspecified renal disease in pregnancy, without mention of hypertension | 646.2 |  |
| Diffuse diseases of connective tissue | 710 | 710 |
| HIV | 042, 079.53,  V08 | 042, 079.53,  V08 |
| Disorders of thyroid gland | 240-246, 648.1 | 240-246 |
| Diabetes | 250.0-250.9, 648.0 | 250.0-250.9 |
| Hypertension | 401-405, 642.0-642.3, 642.9 | 401-405 |
| Pre-eclampsia / eclampsia | 642.4-642.7 |  |
| COPD | 491-492, 494, 496 | 491-492, 494, 496 |
| Asthma | 493 | 493 |
| Cystic fibrosis | 277.0 | 277.0 |
| Acute pulmonary diseases | 480-487, 510-514 |  |
| Chronic pulmonary diseases | 500-508, 515-517 | 500- 508, 515-517 |
| Tuberculosis | 010-018, 647.3 | 010-018 |
| Genital herpes | 054.1 |  |
| Other sexually transmitted diseases | 077.98, 078.88,  079.88, 079.98, 090-099, 647.0- 647.2 |  |
| Antepartum hemorrhage, abruptio placentae, and placenta previa | 641 |  |
| Preterm labor | 644.1, 644.2 |  |
| Late pregnancy | 645 |  |
| Liver disorders in pregnancy | 646.7 |  |
| Polyhydramnios, oligohydramnios / infection of the amniotic cavity | 657, 658.0, 658.4 |  |
| Premature rupture of membranes | 658.1 |  |
| Cord prolapse | 663.0 |  |
| Malposition and malpresentation of fetus | 652 (excl. 652.0, 652.1, 652.5) |  |
| Fetopelvic disproportion/excessive development of the infant | 653, 656.60, 656.61, 656.63 |  |
| Fetal abnormality | 655 |  |
| Intrauterine growth retardation | 656.5, 764 |  |
| Fetal distress | 656.3, 768 |  |
| Multiple pregnancy | 651, V27.2 –V27.9, V31-V37, 761.5 |  |
| Rh isoimmunization | 656.1 |  |
| Maternal conditions affecting fetus or newborn | 760.0, 760.1, 760.3 |  |
| Alcohol or drug dependence/abuse | 303-305; 648.3 (excl. 648.32, 648.34) |  |
| High risk pregnancy | 640, 644.0, V23.0, V23.2, V23.4, V23.5, V23.7, V23.8 |  |
| Assisted fertilization | V26 |  |

* Among the risk factors for cesarean delivery is not taken into consideration dystocia due to poor reproducibility of the definition of this factor and because this diagnosis may reflect subsequent justification of the use of cesarean section.
