# Supplementary material for: Regional Outcome Evaluation Program (P.Re.Val.E.): Reduction of inequality in access to effective health care in the Lazio region of Italy (2012–2015)
Source: PLoS One. 2018 Mar 27;13(3):e0194972. doi: 10.1371/journal.pone.0194972 (PMC5870986; doi:10.1371/journal.pone.0194972)
Supplement: S1 Table — (DOCX) [file pone.0194972.s002.docx]

**Table.** **Association between outcomes and socio economic status (SES) at the beginning and end of the study period (2012 and 2015).**

|  |  | **2012** | | | | |  | **2015** | | | | |
| --- | --- | --- | --- | --- | --- | --- | --- | --- | --- | --- | --- | --- |
|  | **SES** | **N** | **Crude prop. (%)** | **Adj prop.* (%)** | **RR** | **p-value** |  | **N** | **Crude prop. (%)** | **Adj prop.* (%)** | **RR** | **p-value** |
| **STEMI: proportion of patients treated with PCI within 90'** | ***Low*** | 529 | 22.7 | 22.0 | 1 |  |  | 496 | 43.6 | 44.7 | 1 |  |
|  | ***Medium/low*** | 900 | 27.2 | 26.7 | 1.21 | 0.058 |  | 725 | 45.2 | 44.9 | 1.00 | 0.955 |
|  | ***Medium/high*** | 812 | 29.8 | 29.5 | 1.34 | 0.004 |  | 650 | 46.0 | 46.7 | 1.04 | 0.545 |
|  | ***High*** | 427 | 28.1 | 28.6 | 1.30 | 0.024 |  | 365 | 44.7 | 44.7 | 1.00 | 0.998 |
| **Hip fracture: proportion of intervention within 2 days** | ***Low*** | 1080 | 30.2 | 30.1 | 1 |  |  | 898 | 54.3 | 54.9 | 1 |  |
|  | ***Medium/low*** | 1585 | 31.0 | 31.2 | 1.03 | 0.568 |  | 1458 | 52.5 | 52.8 | 0.96 | 0.326 |
|  | ***Medium/high*** | 1564 | 32.9 | 32.8 | 1.09 | 0.146 |  | 1437 | 54.6 | 55.0 | 1 | 0.962 |
|  | ***High*** | 876 | 37.8 | 37.8 | 1.26 | <.001 |  | 833 | 63.8 | 63.9 | 1.16 | <.001 |
| **Proportion of women with primary c-section** | ***Low*** | 6134 | 30.8 | 31.4 | 1 |  |  | 4413 | 26.8 | 26.4 | 1 |  |
|  | ***Medium/low*** | 9667 | 31.6 | 31.5 | 1.00 | 0.911 |  | 7081 | 27.9 | 27.6 | 1.05 | 0.205 |
|  | ***Medium/high*** | 9476 | 31.6 | 30.7 | 0.98 | 0.411 |  | 6842 | 28.5 | 27.3 | 1.04 | 0.318 |
|  | ***High*** | 4963 | 33.9 | 31.9 | 1.02 | 0.615 |  | 3657 | 30.5 | 28.6 | 1.08 | 0.047 |

*Adjusted for:

- STEMI: age, gender, previous myocardial infarction, heart failure, cerebrovascular disease, chronic renal disease, systolic blood pressure at admission.

- Hip fracture: age, gender, diabetes, anemias, other forms of ischemic heart disease, conduction disturbances and arrhythmias, cerebrovascular disease, chronic renal disease.

- Deliveries: maternal age, maternal citizenship, cancer, liver disorders in pregnancy, cardiovascular diseases in pregnancy, Antepartum hemorrhage/abruptio placentae/ placenta previa, Pre-eclampsia/eclampsia, preterm labor, multiple pregnancy, fetopelvic disproportion/excessive development of the infant, fetal abnormality, fetal distress, intrauterine growth retardation, Polyhydramnios/ oligohydramnios/ infection of the amniotic cavity, premature rupture of membranes, cord prolapse, hiv, assisted fertilization.
